# Plasmonic Nanodomains Decorated on Two-Dimensional Oxide Semiconductors for Photonic-Assisted CO_2_ Conversion

**DOI:** 10.3390/ma16103675

**Published:** 2023-05-11

**Authors:** Mohammad Karbalaei Akbari, Nasrin Siraj Lopa, Jihae Park, Serge Zhuiykov

**Affiliations:** 1 Department of Solid-State Sciences, Faculty of Science, Ghent University, Krijgslaan 281/S1, 9000 Ghent, Belgium; nasrin.lopa@ghent.ac.kr (N.S.L.); serge.zhuiykov@ugent.be (S.Z.); 2 Center for Environmental and Energy Research, Ghent University Global Campus, 119-5 Songdomunhwa-ro, Yeonsu-gu, Incheon 21985, Republic of Korea; jihae.park@ghent.ac.kr; 3 Department of Animal Sciences and Aquatic Ecology, Faculty of Bioscience Engineering, Ghent University, Wetenschapspark 1, Bluebridge, 8400 Oostende, Belgium

**Keywords:** two-dimensional materials, 2D plasmonic, Ga_2_O_3_, liquid metals, CO_2_ conversion, carbon capture

## Abstract

Plasmonic nanostructures ensure the reception and harvesting of visible lights for novel photonic applications. In this area, plasmonic crystalline nanodomains decorated on the surface of two-dimensional (2D) semiconductor materials represent a new class of hybrid nanostructures. These plasmonic nanodomains activate supplementary mechanisms at material heterointerfaces, enabling the transfer of photogenerated charge carriers from plasmonic antennae into adjacent 2D semiconductors and therefore activate a wide range of visible-light assisted applications. Here, the controlled growth of crystalline plasmonic nanodomains on 2D Ga_2_O_3_ nanosheets was achieved by sonochemical-assisted synthesis. In this technique, Ag and Se nanodomains grew on 2D surface oxide films of gallium-based alloy. The multiple contribution of plasmonic nanodomains enabled the visible-light-assisted hot-electron generation at 2D plasmonic hybrid interfaces, and therefore considerably altered the photonic properties of the 2D Ga_2_O_3_ nanosheets. Specifically, the multiple contribution of semiconductor–plasmonic hybrid 2D heterointerfaces enabled efficient CO_2_ conversion through combined photocatalysis and triboelectric-activated catalysis. The solar-powered acoustic-activated conversion approach of the present study enabled us to achieve the CO_2_ conversion efficiency of more than 94% in the reaction chambers containing 2D Ga_2_O_3_-Ag nanosheets.

## 1. Introduction

Plasmonic nanostructures have gained an outstanding position in novel photonic technologies during the last decade [1,2]. These photonic structures are capable of tuning and confining light waves at nanoscale dimensions, enabling the generation of the surface plasmon resonance (SPR) phenomenon at plasmonic nanodomains and junctions [3]. The light-matter interactions in plasmonic nanostructures and the following surface plasmon generation actively confine the electromagnetic fields of lights at nanoscale dimensions, triggering a wide range of intricate photonic interactions at plasmonic heterointerfaces [4]. Plasmonic characteristics were observed in a variety of materials, including metals [5], semiconductors [6], and dielectric and conductive oxides [7]. In the conventional metal–semiconductor plasmonic nanostructure, a plasmonic metal (e.g., Au, Ag) is in contact with semiconductor materials [8]. This configuration suffers from radiative loss, high energy dissipation, and a complicated fabrication process [9]. The efficient conversion of visible light through plasmonic nanostructures faces technological challenges, and consequently tremendous efforts have been devoted to enhancing the functionality and efficiency of plasmonic-based nanostructures for functional applications.

Distinguished light–matter interactions and quantum confinement effects are among the main electronic characteristics of 2D materials [10]. These properties provide great opportunities for the development of photonic technologies based on 2D structures. The 2D nanostructures are able to receive various wavelengths of solar radiation from UV to infrared regions [11,12]. Bandgap modulation and heterointerface engineering are the main approaches toward the alteration of electro-photonic characteristics of 2D materials [13,14]. In this area of photonic technology, 2D plasmonic nanostructures represent a novel class of photonic materials with the capability of reception of visible and infrared wavelengths of lights [15]. The formation of hot spots at metal–semiconductor plasmonic heterointerfaces and the following transfer of generated hot electrons to adjacent semiconductor are the well-known mechanisms of visible light harvesting in the metal–semiconductor plasmonic heterostructures [16]. To exploit the functional plasmonic capabilities, various strategies have been developed to control the light–matter interactions. The design of optical nanocavities and photonic junctions are among the main strategies [17]. Through novel design and fabrication technologies, various 2D plasmonic nanostructures have been developed for transparent electronics [18], artificial synaptic technologies [19,20,21,22], biosensing [23], neural interfacing [24], bioinspired technologies [25], and photovoltaics [26]. One of the main forthcoming applications of plasmonic 2D nanostructures is the development of climate-friendly solar-driven technologies for energy generation and environmental applications. The outstanding photonic properties of plasmonic 2D nanostructures accompanied by their physicochemical characteristics provide great opportunities for the conversion of greenhouse gases similar to CO_2_ into value-added byproducts and clean sources of energies [27].

The solar-assisted photocatalysis conversion of greenhouse gases into value-added byproducts is one of the most promising approaches toward the energy-efficient conversion of environmentally hazardous gases [28]. Due to the high thermal stability of C=O bonding, the activation thermal energy for the CO_2_ conversion into intermediate species is considerable (2000 °K), imposing a challenging burden for room-temperature catalytic conversion of CO_2_ [29], though 2D metal-oxide semiconductors are promising catalyst materials for efficient CO_2_ conversion. However, due to their intrinsic high bandgap, the solar-assisted photocatalytic functionalities of them are restricted [30]. Inspired by recent findings, it is believed that plasmonic heterointerfaces can effectively enhance the CO_2_ conversion efficiency [31]. Practical studies have revealed that the visible-light-assisted electron–hole generation at plasmonic interfaces of 2D metal-oxide semiconductors enables plasmonic photocatalysis [32]. The present study developed a novel type of plasmonic 2D hybrid interfaces for efficient room-temperature synergistic CO_2_ conversion. Accordingly, sonochemical functionalization enabled the growth of Ag and Se nanodomains on the surface of 2D Ga_2_O_3_ nanosheets. The 2D Ga_2_O_3_ nanosheets were extracted from the surface oxide films of a gallium-based room-temperature liquid–metal alloy called galinstan. Galinstan is a eutectic alloy of gallium (Ga), indium (In), and stanium (Sn), and therefore it is abbreviated as EGaInSn. In this context, the controlled decoration of 2D semiconductors with plasmonic nanodomains is a highly challenging process. Furthermore, the design of new technologies for efficient CO_2_ reduction is critically important for commercialization and sustainability targets. In this study, the triggering energy for CO_2_ conversion was supplied from combined sources of energies, i.e., simulated solar light and acoustic waves. Consequently, a CO_2_ conversion efficiency of 94.6% was achieved. The process was also accompanied by the generation of O_2_ gas and carbon byproducts. This unique acoustic-activated plasmonic photocatalysis system is expected to address various technical challenges and expectations toward sustainable photonic-assisted conversion of CO_2_ into environmentally friendly byproducts. These 2D plasmonic nanostructures show great potential for development of various types of photocatalyst materials for solar-powered conversion of greenhouse gases into value-added byproducts and clean sources of energy.

## 2. Materials and Methods

**Figure 1:**
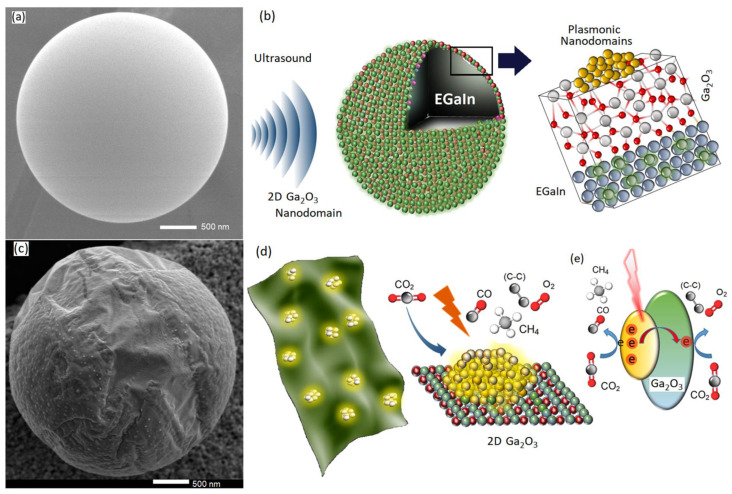
(**a**) SEM image of a singular galinstan NP before sonication treatment accompanied by (**b**) the graphical scheme of sonochemical functionalization and atomic-scale configuration of plasmonic nanodomains grown on a naturally developed 2D Ga_2_O_3_ layer on the surface of galinstan NP. (**c**) A singular galinstan NP after sonochemical functionalization. (**d**) Graphical scheme depicting the delamination of 2D Ga_2_O_3_ film during sonochemical-assisted synthesis and the interaction of CO_2_ gas molecules with 2D Ga_2_O_3_ nanostructure decorated with plasmonic nanodomains. (**e**) Simplified mechanism of transfer of plasmonic hot electrons into adjacent Ga_2_O_3_ film in solar-powered acoustic-assisted CO_2_ conversion process.

To synthesize 2D Ga_2_O_3_ nanostructures, the room-temperature liquid galinstan alloy (EGaInSn) was used in an ultrasonic reactor. The ultrasonic waves effectively dismantled the EGaInSn alloy and distributed the alloy into microsized and finally nanosized particles. The sequential tearing of EGaInSn alloy was accompanied by the separation of 2D surface oxide films of EGaInSn alloy from their parent metal followed by the oxidation of underlayer EGaInSn alloy (Figure 1a–d). These 2D surface oxide films performed as the plasmonic 2D nanostructures in the solar-powered acoustic-activated CO_2_ conversion system of this study. The 2D nanosheets were later extracted and refined after their separation from other products of the sonochemical synthesis process. In detail, after centrifugation, the heavy nanostructures aggregated at the bottom of centrifuge microtubes. The 2D nanosheets were later collected from the remaining liquid after centrifugation. To grow Ag and Se nanodomains on the surface of 2D Ga_2_O_3_ nanosheets of galinstan alloy, different solutions were prepared by the sonication of AgCl_4_, and SeCl_4_ in ethyl alcohol anhydrous fluid containing galinstan alloy. The slurries were probe-sonicated in the ionic solutions with different concentrations of AgCl_4_, and SeCl_4_ (0.1 and 1.0 μmol/L) for an hour (Figure 1b). For material characterization, the 2D Ga_2_O_3_ nanosheets were extracted, dried in controlled atmosphere and then investigated by various methods. A micro-Raman spectrometer (micro-Raman HORIBA Lab Ram ARAMIS) equipped with λ = 320 nm and 280 nm lasers was employed to extract the Raman and photoluminescence (PL) spectra of synthesized 2D nanostructures. To this end, the solutions containing 2D nanostructures were drop-casted on the Si/SiO_2_ substrate and then dried in a controlled atmosphere. The individual 2D nanostructures were selected under microscopy and the PL spectra were collected for 15 s. X-ray photoelectron spectroscopy (XPS) was later employed for analysis of surface composition of 2D nanostructures after functionalization (XPS-Scientific Theta Probe). An X-ray diffractometer (XRD, Bruker D8) was employed to identify the crystalline structure of as-grown nanodomains on 2D Ga_2_O_3_ nanosheets. Field-emission SEM (FESEM, JEOL 7800F), and high-resolution TEM (TEM, JEM-2100Plus) were employed to investigate the structural characteristics of synthesized nanostructures. An atomic force microscope (AFM Park System NX 10) was used to analyze the surface morphology of synthesized 2D nanosheets. In situ Fourier-transform infrared spectroscopy (FTIR-Nicolet iS5) with a predesigned gas chamber was employed to monitor the in situ reaction of CO_2_ with 2D Ga_2_O_3_. The efficient acoustic-activated CO_2_ reduction technique was developed by using 2D Ga_2_O_3_ nanosheets in an ultrasonic-assisted conversion reactor. In our setup, the suspension of 2D Ga_2_O_3_-Ag and 2D Ga_2_O_3_-Se nanostructures (50 gr/L) was agitated by ultrasonic waves in a quartz chamber containing ethyl alcohol anhydrous. A xenon lamp (DY. TCH) was used to simulate the solar radiation during reactions. The high-purity CO_2_ (99.9%) was introduced into a 30 mm reactor with input rate of ~5 sccm at 20 °C. The composition of extracted gases from the conversion chamber was monitored by a high-precession CO_2_/O_2_ gas sensor (Oxybabay M+ CO_2_/O_2_). The measurement limit of sensors was 10 ppm. The byproducts of the CO_2_ conversion process were later extracted and examined by TEM. To this end, TEM grids were immersed into the top layer of extracted liquid containing byproducts of CO_2_ conversion process and were dried later in a vacuum chamber.

## 3. Results

### 3.1. Synthesis of 2D Ga_2_O_3_ Nanosheets with Plasmonic Nanodomains

Generally, 2D materials are synthesized by different techniques. Mechanical exfoliation [33], chemical vapor deposition [34] and atomic layer deposition [35] are among the main commercially available techniques for fabrication of 2D nanostructures. Mechanical delamination of 2D layers of brittle structures in fluid medium is one of the most efficient techniques for large-scale synthesis of 2D materials [36]. The high surface tension of room-temperature liquid metal galinstan effectively suppresses the fragmentation of this alloy into ultrafine nanoparticles (NPs). Ultrasonic waves provide strong mechanical forces for functional applications [37]. Here, we developed a new concept to synthesize 2D Ga_2_O_3_ nanostructures from a gallium-based alloy. The ultrasonic waves were able to separate the natural surface oxide film of an EGaInSn alloy from its parent alloy (Figure 1b,c). The outward explosion and also the inward implosion of bubbles during sonication produce microjets and shock waves at ultrasound speeds that also accelerated the moving particles inside the liquid medium at several hundred meters per second (m/s) [38]. The high-energy/high-speed microjets provide strong shear forces for drastic mechanical fragmentation of materials, known as the sonofragmentation process [30,31,32,33,34,35,36,37,38,39,40,41]. Consequently, the acoustic-activated energy can effectively supply the driving force for sequential delamination of 2D surface oxide Ga_2_O_3_ nanosheets from their parent EGaInSn alloy [42]. Apart from the mechanical delamination of surface oxide 2D nanosheets, the ultrasonic waves are capable of synthesis of various types of nanostructures. The generated hot-spot regions in the ultrasonic process carry a high temperature (5000 K) and pressures (1000 atm) providing the supplied energies with a magnitude of 13 eV [43]. The high-energy particle collisions, plasma generation [43,44] and nuclear fusion [45] are observed in hot-spot regions generated during ultrasonication. Cooperative interactions between the precursors and ionic species inside acoustic bubble cores result in the synthesis of a wide range of nanostructured materials. The synthesis driving force is provided by the extremely unusual conditions in the core of hot-spot regions [46,47]. In detail, the diffusion of precursors into hot-spot cores is accompanied by the interaction of precursors and other components in the reaction medium, resulting in the synthesis of new nanostructures. The synthesized hot materials in the core of hot spots suddenly quench at the rate of 10^10^ K/s [48] after the eruption of magma matter into the surrounding fluid environment. The generated thermal shocks enable the immediate growth of various types of nanostructures [49]. Chemical reactions may also occur outside hot-spot regions due to interactions between ionic species and scattered radicals. In this mechanism, the synthesis conditions are free from the extraordinary physical states of hot spots. Therefore, the synthesized materials have properties similar to conventional nanostructures [46]. The control of precursor concentration and the reaction parameters fundamentally affect the sonochemical reactions and prompt the synthesis of new materials with stabilized growth directions. The sonochemical-assisted synthesis of 2D Ga_2_O_3_ in metal-ionic solutions enables the growth of crystalline nanostructures on the surface of 2D materials (Figure 1d). Here, the 2D Ga_2_O_3_ nanosheets act as the nucleation cites for growth of various nanostructures, including metallic nanodomains. The acoustic-assisted decoration of 2D nanostructures with crystalline nanodomains enables the development of 2D hybrid plasmonic interfaces. The plasmonic nanodomains decorated on 2D Ga_2_O_3_ nanosheets alter the electronic properties and energy band alignment at the metal–semiconductor plasmonic heterointerfaces. It is expected that the visible-light properties of these plasmonic 2D structures favor the solar-powered physical and chemical reactions during CO_2_ conversion through transfer of plasmonic-generated hot carriers into surrounding reaction locations (Figure 1d,e). The mechanism of CO_2_ conversion will be discussed later.

### 3.2. Characterization of 2D Ga_2_O_3_ Nanosheets

**Figure 2:**
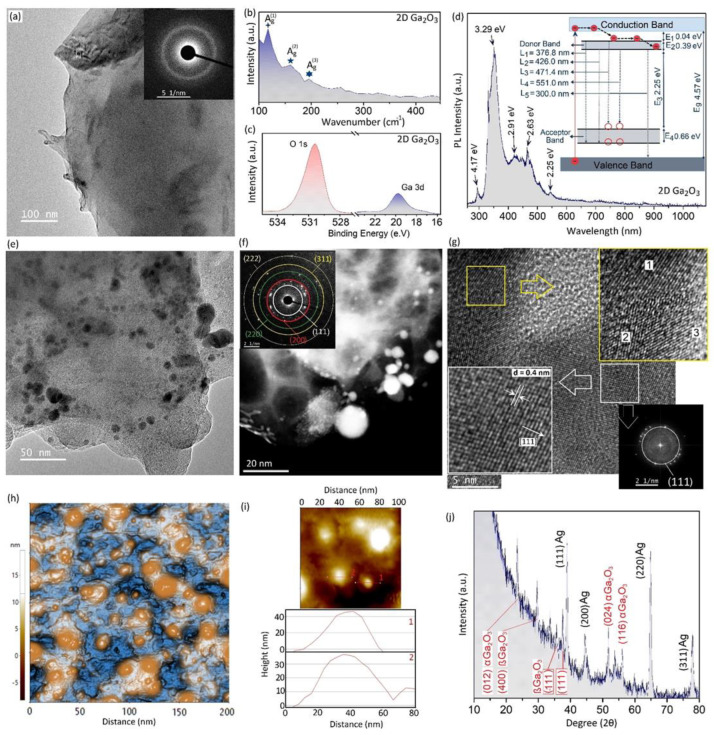
(**a**) TEM image and corresponding SAED pattern of pristine 2D Ga_2_O_3_ nanosheets; (**b**) Raman spectra and (**c**) XPS characteristics of pristine 2D Ga_2_O_3_ nanosheets; (**d**) PL spectra of pristine 2D Ga_2_O_3_ nanosheets; (**e**) TEM image of 2D Ga_2_O_3_ film with Ag nanodomains and its corresponding (**f**) dark-field TEM image accompanied by its SAED pattern; (**g**) HRTEM images of Ag nanodomains and the corresponding images of crystalline planes of Ag accompanied by their corresponding FFT patterns; (**h**) AFM image of surface of 2D Ga_2_O_3_ film with Ag nanodomains on the surface; (**i**) AFM thickness profile of Ag NPs; (**j**) XRD patterns of 2D Ga_2_O_3_-Ag nanostructures.

We initially investigated the properties of pristine 2D Ga_2_O_3_ nanosheets by characterization techniques. The TEM image and its corresponding SAED patterns of pristine 2D Ga_2_O_3_ nanosheets depict the halo rings confirming the disordered structural characteristics of these 2D nanostructures (Figure 2a). Therefore, it is expected that pristine 2D Ga_2_O_3_ has an amorphous nature. The thickness of pristine Ga_2_O_3_ nanosheets was in the range of a few nanometers to tens of nanometers (few cases). The lateral dimensions of 2D nanosheets were in the range of hundreds of micrometers. The following studies by Raman spectroscopy showed the characteristic peaks of $A_{g}^{1}$ $A_{g}^{2}$ and $A_{g}^{3}$, respectively, at 114 cm^−1^, 166 cm^−1^ and 199 cm^−1^ (Figure 2b). These peaks are related to the vibrational mode of ß-Ga_2_O_3_ structure [50]. The following XPS studies evidently showed the Ga 3d peak at 19.9 eV. The distinguished O 1s peak with central position of 529.6 eV is the characteristic peak of oxygen atoms, which is bonded to Ga atoms in Ga_2_O_3_ structures (Figure 2c). We further investigated the PL spectra of pristine 2D Ga_2_O_3_ nanosheets with a UV laser with λ = 280 nm wavelength. Figure 2d shows the typical PL spectra of pristine 2D Ga_2_O_3_ nanosheets. The PL spectra are characterized by several peaks. Two sharp peaks centered at ~300 nm and ~376 nm of the UV region and two peaks centered at ~426 nm and ~471.4 nm in blue regions were observed. A singular peak was also detected at 551.04 nm in the green region of spectrum. The relative intensity of UV luminescence is considerably higher than that of the peaks at blue and green regions (Figure 2d). The PL emission can be attributed to transition of electrons from the donor band to the acceptor and valence bands of 2D Ga_2_O_3_ [51]. Due to the disordered nature of pristine 2D nanosheets, it is expected that nonradioactive recombination occurs during the PL emissions [52]. In Figure 2d, the major emission bands are detected at 376.8 nm (L_1_, 3.29 eV), 426.0 nm (L_2_, 2.91 eV), 471.4 nm (L_3_, 2.63 eV), 551.0 nm (L_4_, 2.25 eV), and an individual minor peak at ~300.0 nm (L_5_, 4.17 eV). The detection of UV emission in pristine 2D Ga_2_O_3_ can be explained by a model that suggests the electrons and holes can be de-trapped due to photoexcitation [52,53]. The migration and incidence of these electron–hole pairs can form self-trapped excitons. These excitons can recombine and emit UV photons [52,53]. A similar mechanism for UV emission was previously reported for single crystal and nanostructured ß-Ga_2_O_3_ [52,53]. The UV-green emission in non-doped Ga_2_O_3_ structure can be attributed to the recombination of an electron on the donor band of Ga_2_O_3_ with another hole formed in the acceptor band of this material [54]. The oxygen vacancies and Ga^2+^ form a donor band, while the acceptor band can be formed by the gallium vacancy and pairs of gallium–oxygen vacancy [52]. A simplified model is extracted from the PL spectra of 2D pristine Ga_2_O_3_ nanosheets and is shown in Figure 2d. The donor band (E_1_) is located 0.04 eV below the conduction band minimum (CBM), which is attributed to the formed oxygen vacancies [52,54]. The electron photoexcitation from conduction band to valence band is accompanied by electron relaxation where the electron can freely move from conduction band to donor band before the occurrence of radiative recombination. The following electron–hole recombination between donor and acceptor bands yields in the generation of UV-green emission in the PL spectra of pristine Ga_2_O_3_ nanosheets (Figure 2d). We further analyzed different energy levels in the bandgap of pristine 2D Ga_2_O_3_ nanosheets, and the results are presented in the following lines and depicted in Figure 2d:

E (L_1_) − E (L_2_) = E_2_ = 0.39 eV (1)

E (L_3_) − E (L_4_) = E_2_ = 0.39 eV (2)

E (L_1_) − E (L_3_) = E_4_ = 0.66 eV (3)

E (L_2_) − E (L_4_) = E4 = 0.66 eV (4)

E_g_ (4.57 eV) − E_1_ − E_2_ = 4.17 eV (5)

These results are employed to depict the energy band level for pristine Ga_2_O_3_ nanosheets (Figure 2d). The calculated value in (5) is equal to 4.17 eV, which is consistent with the energy level of detected minor peaks at ~300.0 nm. This emission is related to the recombination of electrons in the donor band with the holes in valence band edge [52].

The functionalization of 2D Ga_2_O_3_ nanosheets with plasmonic nanodomains was successfully achieved by sonochemistry-assisted technique. Figure 2e shows transparent 2D Ga_2_O_3_ nanosheets with Ag nanodomains on its surface. The silver NPs can be decorated on the Ga_2_O_3_ surface oxide of galinstan alloy either before or after delamination of 2D nanosheets from their parent alloy. It is believed that the 2D Ga_2_O_3_ nanosheets act as the nucleation cites for Ag NPs. Furthermore, the gallium on the surface of liquid–metal alloy can also take part in a galvanic reaction where the Ga^0^ atoms can be replaced by the ionic Ag^+^ according to the following Equation (6) [55]:

Ga^0^ + 3Ag^+^ → Ga^3+^ + 3Ag^0^ (6)

Ag nanodomains with average size of less than ~20 nm were grown during sonochemical synthesis on 2D Ga_2_O_3_ nanosheets. The TEM dark-field image (Figure 2f) shows the distribution of Ag NPs on 2D nanosheets. The distribution of Ag nanostructures on surfaces of 2D nanosheets confirms that these nanodomains grew independently during sonication. The SAED patterns of the same position on 2D nanosheets were collected and are presented in Figure 2f. The diffraction patterns show the growth of various crystalline planes of Ag i.e., (111), (200), (220), (331) and (222) [56]. Figure 2g presents an HRTEM image of two adjacent Ag NPs, their atomic arrangements and corresponding high-resolution fast Fourier-transform (FFT) pattern of an individual region. The HRTEM image in top-right section of Figure 2g demonstrates the heterojunction between three individual nanodomains with different crystalline growth directions. These regions are marked with numbers 1, 2 and 3. It further confirms that Ag nanodomains nucleated and grew individually at different locations on 2D Ga_2_O_3_ nanosheets without any preferential growth direction. The more detailed observations in Figure 2g (bottom-left) show the crystalline planes with corresponding distance of 0.4 nm. This interlayer distance can be attributed to the planar space between (111) planes of crystalline Ag nanodomains, which is also confirmed by the results of FFT studies (Figure 2g) [56]. The following study on the 2D Ga_2_O_3_ nanosheets by AFM shows the morphology of 2D Ga_2_O_3_ nanosheets and Ag nanostructures on it (Figure 2h). The Ag nanostructures on 2D nanosheets can be distinguished vividly. The thickness profiles of two individual Ag NPs are measured and presented in Figure 2i. A typical Ag nanostructure has the dimension of 20 nm (Figure 2i). The AFM studies provide valuable information about the morphology and surface characteristics of 2D Ga_2_O_3_ nanosheets and Ag nanodomains. Ag nanodomains grew uniformly on the surface of 2D Ga_2_O_3_ nanosheets. The XRD pattern of functionalized 2D Ga_2_O_3_-Ag nanosheets is depicted in Figure 2j. The XRD characteristics of crystalline planes of (111), (200), (220) and (311) of Ag are found, which are in agreement with JCPDS 04-0783 [56]. The other characterized peaks can be attributed to the crystalline planes of α- and ß-Ga_2_O_3_. It confirms the growth of crystalline phases of gallium oxide during the sonochemical synthesis process.

Material characterization studies also further confirmed the crystalline nature of synthesized Ag nanodomains decorated on the surface of 2D Ga_2_O_3_ nanosheets.

**Figure 3:**
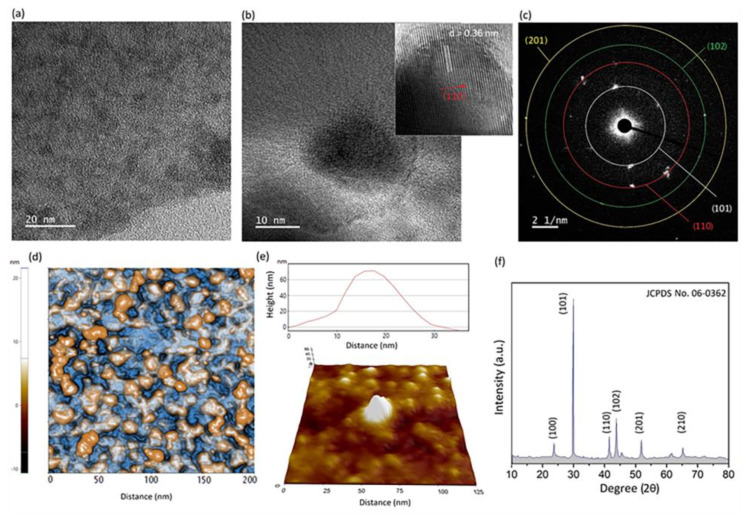
(**a**) TEM image of 2D Ga_2_O_3_ nanosheets with Se nanodomains and its corresponding (**b**) TEM image of a singular Se nanodomain accompanied by corresponding HRTEM image and (**c**) SAED patterns of crystalline planes of Se; (**d**) AFM image of surface of 2D Ga_2_O_3_ film with Se nanodomains grown on the surface; (**e**) AFM thickness profile of singular Se nanodomain; (**f**) XRD patterns of 2D Ga_2_O_3_-Se nanostructures.

We further characterized the 2D Ga_2_O_3_-Se nanosheets. Figure 3a depicts a TEM image of 2D Ga_2_O_3_-Se nanosheets. The ultrasonic waves create extreme localized hot spots that enable complex physicochemical reactions. At the Ga_2_O_3_ surface, the interfacial reactive wetting is enhanced due to intensified turbulence, which consequently prompts a high level of ion mobility and mass transfer from the Se ionic regions to the surface of 2D Ga_2_O_3_. Generally, Se forms in amorphous, metallic and crystalline polymorphs [57]. Low melting (217 °C) and low glass transition (31 °C) temperatures facilitate the synthesis of amorphous Se at room temperature that can be transformed into trigonal Se in the conventional thermal synthesis methods [58]. The ultrasonic waves enabled the synthesis of Se nanostructures in unusual non-thermodynamic conditions [59]. At the initial stage of sonication treatment of the samples in Se containing ionic solution, the sequential decomposition of SeCl_4_ into Se_2_Cl_2_ and HCl leads to the rapid nucleation of Se nanostructures on nucleation sites of 2D Ga_2_O_3_. The Se atoms tend to form mono-Se particles on 2D Ga_2_O_3_ structures due to the elevated reactive wetting. Upon the increase in sonication time, Se nanostructures actively nucleate and grow on the surface of 2D nanosheets, which later agglomerate in the form of Se clusters and nanodomains. Figure 3b depicts the Se nanocrystalline structure with its corresponding HRTEM image. The HRTEM image shows the crystalline interlayer distance of 0.39 nm in the crystalline direction of (110). The following study on the SAED patterns of synthesized Se nanodomains confirmed the presence of crystallographic planes of (101), (110), (102) and (201) attributed to the crystalline Se nanostructure (Figure 3c) [60,61]. The flowing AFM studies on the surface morphology and characteristics of 2D Ga_2_O_3_-Se nanosheets depict the distribution of both singular and agglomerated Se nanostructures on the surface of 2D Ga_2_O_3_ nanosheets (Figure 3d). In some rare cases, the Se nanodomains formed clusters with an average size of 50 nm (Figure 3d). However, in most typical examples, the size of the Se nanodomains was less than 20 nm. Figure 3e provides the thickness profile of a Se nanodomain and its 3D surface morphology. We further investigated the crystalline state of synthesized Se nanodomains via XRD. To this end, 2D Ga_2_O_3_ nanosheets were sonicated in a solution containing 1 μmol SeCl_4_ in ethyl alcohol anhydrous. The extracted nanosheets were later dried in controlled atmosphere and tested. It was observed that the surfaces of samples were covered with a red-tinted Se layer. The XRD results confirmed the presence of planes of (100), (101) (110) 102) and (201) related to the crystalline plane of Se (JCPDS 06-0362) [62]. These results further confirmed the polycrystalline nature of sonochemically synthesized Se nanostructures.

**Figure 4:**
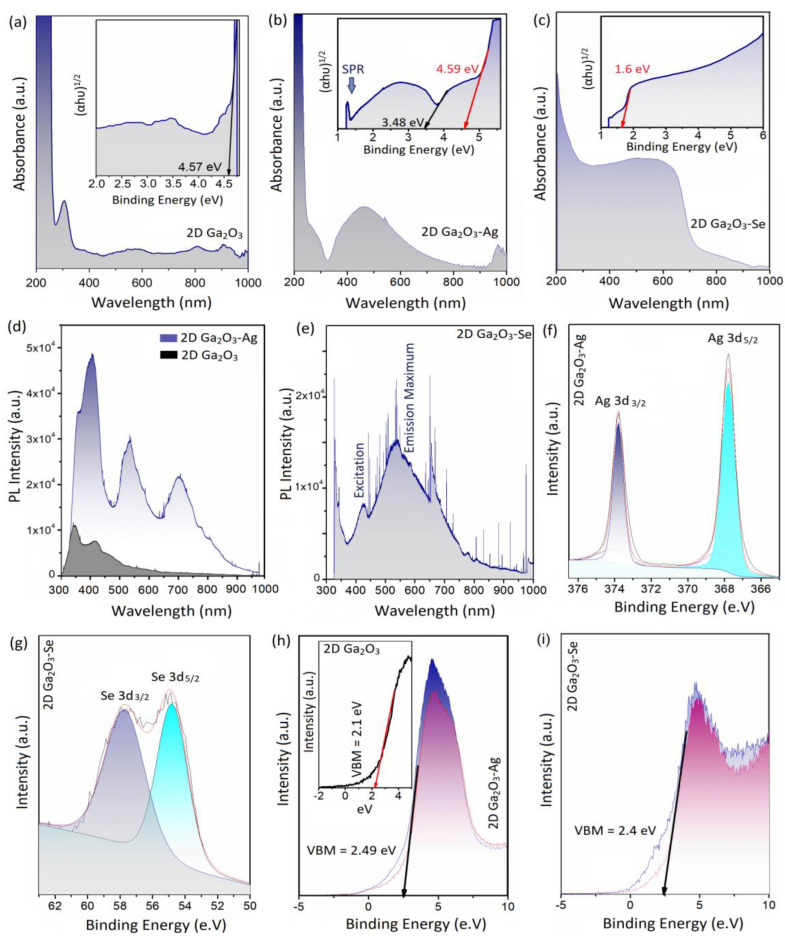
(**a**) UV-vis absorbance spectra of 2D Ga_2_O_3_ nanosheets accompanied by corresponding bandgap calculation (Inset graph); (**b**) UV-vis absorbance spectra of 2D Ga_2_O_3_-Ag nanostructure accompanied by corresponding bandgap calculation (Inset graph); (**c**) UV-vis absorbance spectra of 2D Ga_2_O_3_-Se nanostructure accompanied by corresponding bandgap calculation (inset graph); (**d**) PL spectra of 2D Ga_2_O_3_-Ag nanostructure; (**e**) PL spectra of 2D Ga_2_O_3_-Se nanostructure; (**f**) Ag 3d XPS spectra of 2D Ga_2_O_3_-Ag nanostructure; (**g**) Se 3d XPS spectra of 2D Ga_2_O_3_-Se nanostructure (**h**) and VBM of 2D Ga_2_O_3_ and Ga_2_O_3_-Ag nanostructure; (**i**) VBM of 2D Ga_2_O_3_-Se nanostructure.

UV-vis and PL spectroscopy are functional methods for investigation of photonic properties of 2D plasmonic structures. The bandgap measurements of 2D Ga_2_O_3_ and Ga_2_O_3_-Ag nanosheets gave interesting information on the electronic characteristics of them. The typical absorbance spectra (UV-vis test) of 2D Ga_2_O_3_ and Ga_2_O_3_-Ag nanosheets are presented in Figure 4a,b, respectively. The calculated (inset in Figure 4a) bandgap of ~4.57 eV can be attributed to the bandgap of pristine 2D Ga_2_O_3_ nanosheets. The typical absorbance spectrum of 2D Ga_2_O_3_-Ag nanostructures is also demonstrated in Figure 4b. The following calculations showed two individual bandgaps of ~4.59 eV and ~3.48 eV. The higher bandgap (4.59 eV) can be attributed to semiconducting characteristics of 2D Ga_2_O_3_ nanosheets. The Gaussian peak at 400 nm~500 nm was observed in absorption spectra of 2D Ga_2_O_3_-Ag nanosheets (Figure 4b). Similar observations were also reported in a study of UV-vis characteristics of synthesized silver nanoparticles [63]. This peak is attributed to the optical direct bandgap of silver nanoparticles, which was 3.48 eV in the present study. This number is mostly in agreement with the previously reported bandgap of silver NPs [63]. Furthermore, the plasmonic characteristics of Ag NPs can be responsible for the absorption peak at vicinity of 400 nm [64]. The strong plasmonic resonance absorption peak at the vicinity of λ = 410~430 is one the main characteristics of Ag nanoparticles known as the plasmonic resonance absorption peak [65,66]. The location and intensity of the plasmonic resonance peak are affected by the dimensions of silver NPs. It was shown that the plasmonic resonance absorption of Ag NPs with particle sizes of 10–20 nm occurs at the vicinity of λ = 400 nm [66]. It was reported that an increase in Ag particle size results in the increased scattering, and therefore the plasmonic resonance absorption peak broadened and shifted toward higher wavelengths, known as red shift of light [67]. The plasmonic resonance absorption peaks in this study were due to the presence of ultrafine Ag plasmonic nanoparticles decorated on 2D Ga_2_O_3_ nanosheets. The evidence of occurrence of surface plasmon resonance (SPR) was also observed by the detection of the broad peak at λ~500 nm related to the plasmonic characteristics of Ag nanodomains [66]. The photonic local field enhancement and SPR occurred at heterointerfaces between Ag nanodomains at 2D Ga_2_O_3_. Consequently, the SPR characteristic peaks appeared at absorption spectra of Ga_2_O_3_-Ag heterointerfaces. In practice, the refractive index of the adjacent environment considerably affects the extinction spectrum [67]. A high refractive index for materials similar to 2D Ga_2_O_3_ nanosheets can cause a red shift in the location of the extinction peak. The Ag nanodomains of this research are in contact with 2D Ga_2_O_3_ and air; therefore, the transfer of resonance adsorption and SPR peaks to higher wavelengths is expected in absorbance spectra of 2D Ga_2_O_3_-Ag nanostructures. The study on UV-vis spectra of 2D Ga_2_O_3_-Se nanosheets showed a bandgap of 1.6 eV, which is the characteristic bandgap of Se crystalline nanostructures (Figure 4c) [68].

In the PL spectroscopy, it is possible to focus on an individual 2D Ga_2_O_3_ nanosheets and collect the PL spectra of 2D nanostructures. Figure 4d depicts the PL spectra of pristine 2D Ga_2_O_3_ and 2D Ga_2_O_3_-Ag nanostructures. The PL spectra of 2D Ga_2_O_3_-Ag nanosheets demonstrate three individual peaks at wavelengths of 380 nm, 550 nm, and ~700 nm. These peaks originate from the PL characteristics of Ag nanoparticles enhanced by the strong local electric field of Ag nanodomains [69]. The peak around 340–400 nm is attributed to the interband radiative transitions in Ag nanoparticles [69]. The shoulder in the PL spectrum at 345 nm is close to the maximum of the PL band of bulk silver that occurs at 330 nm. This peak is attributed to the direct radiative interband recombination between the electrons in the conduction band and holes in the valence band of silver that had been scattered to momentum states (less than the Fermi momentum). The intense peak at 390 nm (3.2 eV) is quite close to the intraband absorption edge of bulk silver [69]. The recognized red shift in PL peaks of these NPs (compared with bulk silver) is due to the coupling of the exciting and emitted photons with SPR [69]. The peak in the vicinity of 450–500 nm is also close to the SPR of silver NPs. Therefore, this peak originated from the low-energy wings of the intraband PL peak and is enhanced by the SPR effects. Similar results were also observed in a study of PL spectra of Ag NPs, where the PL bands were located in the vicinity of SPR peaks [69]. The typical PL spectra of 2D Ga_2_O_3_-Se nanostructures are presented in Figure 4e. The PL studies on the same sample demonstrate two peaks at the λ = 340 nm, and λ = 427, and another broad peak centered at λ = 558 nm. These peaks (λ = 427 nm and λ = 558 nm) are respectively related to the excitation and emission peaks of Se nanodomains [68]. Another peak at λ = 650 nm was also detected. The broad peak at λ = 558 nm and the peak at λ = 650 are attributed to the excitation of surface plasmon of Se nanostructure. The red tint of synthesized 2D Ga_2_O_3_-Se nanostructures has been ascribed to the corresponding excitation of the surface plasmon resonance of Se nanostructure [70]. It appeared that the heterointerfaces between of 2D Ga_2_O_3_ nanosheets and Se nanodomains possessed a high level of blue luminesce peak. The PL spectra of 2D Ga_2_O_3_-Se is quite interesting, since the peaks of green and red luminescence are stronger than those of UV luminescence peaks. The Se nanostructures are well known for their photonic, photovoltaic, and semiconducting properties; therefore, these nanostructures can extensively contribute to the performance of 2D Ga_2_O_3_ nanosheets for photonic applications. It is worth mentioning that UV luminescence is affected by the recombination at the surface states by the surface characteristics of materials [71]. The dangling bands and other surface impurities act as the recombination cites for carriers. The presence of metallic nanodomains on the surface can enhance the density of permanent surface defects and therefore influence the PL intensity of 2D Ga_2_O_3_ nanosheets [71]. In blue and red luminescence phenomena, both electrons and holes are trapped in donor and acceptor levels within the bulk structure of materials. In contrast, it is expected that the photoexcited conduction band electrons diffuse to the surface of 2D nanosheets and then are trapped in the structural defects at surface of the Ga_2_O_3_ nanosheets decorated with crystalline nanoparticles. This phenomenon increases the possibility of non-radiative recombination and therefore affects the intensity of UV photoluminescence intensity [71].

### 3.3. Solar-Powered Acoustic-Activated CO_2_ Conversion

**Figure 5:**
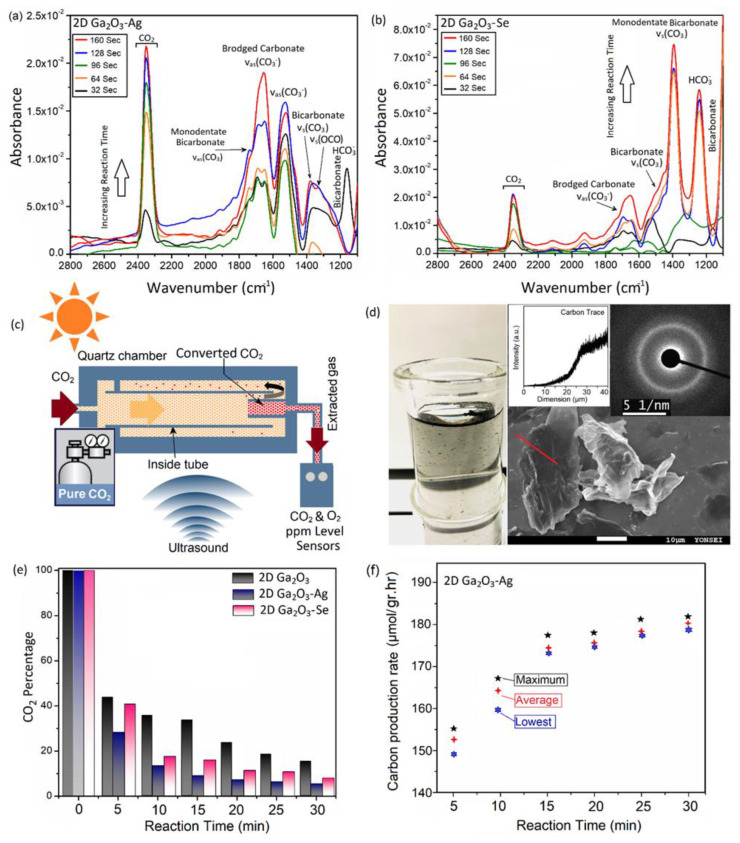
(**a**) FTIR patterns of CO_2_ absorption on 2D Ga_2_O_3_-Ag nanostructure after exposure to dynamic CO_2_ gas; (**b**) FTIR patterns of CO_2_ absorption on 2D Ga_2_O_3_-Se nanostructure; (**c**) graphical scheme of acoustic-activated solar-powered CO_2_ conversion setup; (**d**) collected carbon byproducts after triboelectric–photocatalytic conversion of CO_2_ accompanied by corresponding SEM image and SAED patterns; (**e**) CO_2_ content after conversion vs. reaction time for pristine 2D Ga_2_O_3_, 2D Ga_2_O_3_-Ag and 2D Ga_2_O_3_-Se nanostructures (the solution contains 1 μmol/L of Ag or Se during sonochemical synthesis of 2D nanosheets); (**f**) carbon production rate vs. the reaction time in triboelectric–photocatalytic CO_2_ conversion process.

The gas adsorption on catalyst surface is one of the main characteristics of synthesized 2D materials. We investigated the in situ adsorption of CO_2_ gas on the surface of 2D Ga_2_O_3_ nanosheets via FT-IR. FT-IR spectra were recorded in absorbance mode on a Nicolet spectrometer equipped with a quartz gas chamber with KBr windows. To this end, a quartz chamber containing 2D Ga_2_O_3_ nanostructures was used in the FTIR machine and the adsorption characteristics were monitored sequentially at different stages of reactions. Figure 5a depicts the dynamic absorbance spectra of 2D Ga_2_O_3_-Ag nanostructures in sealed quartz chamber containing highly pure dry CO_2_ gas at room temperature. These results clearly showed that the adsorption of CO_2_ on the surface of 2D Ga_2_O_3_ nanosheets was accompanied by the formation of number of distinct carbonate and hydroxyl species on the surface. The detection and assignment of these carbonate groups are based on the last studies on CO_2_ adsorption process on Ga_2_O_3_ polymorphs [72]. A closer look at Figure 5a revels that the increase of reaction time leads to the formation of distinct signals of CO_2_ at ~2350 cm^−1^. Furthermore, the intensities of corresponding absorbance peaks of other carbonate groups also increased (Figure 5a). In detail, the intensities of the adsorption peaks at 1165 cm^−1^ (bicarbonate HC$O_{3}^{-}$), 1335 cm^−1^ (bicarbonate *v*_s_(OCO)), 1377 cm^−1^ (bicarbonate *v*_s_(CO_3_)), 1528 cm^−1^ (*v_a_*_s_(C$O_{3}^{-}$)), 1656 cm^−1^ (bridged carbonate *v_a_*_s_(C$O_{3}^{-}$)), and 1793 cm^−1^ (monodentate bicarbonate (*v_a_*_s_(CO_3_)) increased vividly during interaction of 2D Ga_2_O_3_-Ag nanostructures with CO_2_ gas [73]. These results confirm the adsorption of CO_2_ and also the formation of various carbonate groups on the surface of 2D Ga_2_O_3_-Ag nanostructures. The detection of bicarbonate and monocarbonate groups is correlated with the adsorption of hydroxyl groups on the surface of 2D Ga_2_O_3_ nanosheets. It is realized that the fundamental stage in CO_2_ conversion is the chemisorption of CO_2_ molecules on the 2D Ga_2_O_3_ nanosheets via insertion into a basic hydroxyl group on the surface of 2D metal oxide nanosheets. This process leads to the formation of bicarbonate groups [73,74]. We further investigated the FTIR absorbance spectra of 2D Ga_2_O_3_-Se nanostructures under the dynamic exposure to the CO_2_ gas. A typical dynamic absorbance FTIR spectra of 2D Ga_2_O_3_-Se nanosheets demonstrates continues increase in the intensity of bicarbonate peaks (Figure 5b). A comparison between Figure 5a and b shows that the peak intensities of bicarbonate and monodentate bicarbonate groups on the surface of 2D Ga_2_O_3_-Se nanostructures are tangibly stronger than those of 2D Ga_2_O_3_-Ag structures. A possible explanation can be attributed to the effect of Se on the absorption of hydroxide groups on the surface. Se actively reacts with the hydroxyl groups in atmosphere. It can explain the higher intensity of absorption peaks of carbonates groups on the 2D Ga_2_O_3_-Se nanostructures, compared with that of 2D Ga_2_O_3_-Ag nanosheets. Therefore, the in situ FTIR results confirmed the time-dependent increasing capability of synthesized 2D nanostructures for chemisorption of CO_2_ molecules. We further investigated the CO_2_ conversion efficiency of these 2D nanostructures. Regarding the distinguished photonic and plasmonic properties of 2D Ga_2_O_3_-Ag and 2D Ga_2_O_3_-Se nanostructures, the solar-powered CO_2_ conversion capacity of these 2D nanostructures is investigated in the next section. Furthermore, we used the ultrasonic generators to combine the effects of acoustic waves and solar radiation for synergistic conversion of CO_2_ gas molecules.

Acoustic-activated CO_2_ reduction has recently been developed for efficient conversion of CO_2_ into value-added byproducts in the presence of gallium and Ga_2_O_3_ based catalyst nanostructures [75]. Driven by effects of acoustic energies, the efficient CO_2_ conversion was achieved when gallium-based nanoparticles were used as catalyst materials [75]. It is confirmed that the strong mechanical triboelectric forces efficiently provide the energy for CO_2_ conversion [75]. The sonochemical triboelectric forces contribute the interfacial phenomena in the heterointerfaces of catalyst materials. In the present study, we innovatively used both mechanical energy of acoustic waves and the solar-powered plasmonic effects to enable the efficient conversion of CO_2_. To this end, we designed a transparent quartz reactor was submerged in an ultrasonic bath (Figure 5c). The solar-powered energy was supplied by a solar simulator xenon lamp at the power of 250 W and output wavelength of 350–1100 nm (Figure 5c). It is worth mentioning that we investigated the amounts of CO_2_ and O_2_ in the input and exhaust gases to show the efficiency of our conversion technique. Therefore, precise CO_2_/O_2_ sensors with 10 ppm measurement limit were employed to monitor the input and exhaust gases. It should be mentioned that chamber was totally sealed and the process of extraction of exhaust products was done by the collection of output gases via a sealed gas box, mounted on the nozzle of the sonication system. After the CO_2_ conversion process, the solid byproducts of reactions were found suspended on the quartz vials (Figure 5d). After filtration of byproducts, the samples were investigated by SEM and TEM. The SEM image, EDX line analysis and corresponding SAED patterns of TEM studies are presented in Figure 5c. The EDS analysis confirmed that the extracted byproducts are composed of 83 wt.% carbon. The TEM studies showed halo rings in SAED patterns confirming the amorphous nature of carbon byproducts (Figure 5d). The exhaust gases were measured by the ppm level CO_2_ and O_2_ sensors to calculate the CO_2_ conversion efficiency of system. Figure 5e depicts the remaining CO_2_ content after the conversion process. It is necessary to mention that the exhaust gas is able to circulate in the reaction chamber. Therefore, the CO_2_ content was measured after 5, 10, 15, 20, 25 and 30 min from the start of cyclic conversion. It can be observed that the CO_2_ percentage decreased considerably in both 2D Ga_2_O_3_, 2D Ga_2_O_3_-Ag, and 2D Ga_2_O_3_-Se samples under solar radiation and mechanical agitation of acoustic waves. Results show that the triboelectric and photocatalytic mechanisms can lower the CO_2_ content to less than 20% after 30 min of cyclic reactions in the chamber containing pristine 2D Ga_2_O_3_ nanosheets. However, the effect of solar radiation on photonic conversion of CO_2_ became more visible when the 2D Ga_2_O_3_-Ag and 2D Ga_2_O_3_-Se nanosheets were employed in the reaction chamber. In an optimized case, the CO_2_ content in exhaust gases declined to the values less than 5% after 30 min of sequential conversion in the chamber containing 50 gr/L concentration of 2D Ga_2_O_3_-Ag nanosheets under 250 W radiation of solar-light simulator. Therefore, 94.6% CO_2_ conversion efficiency was achieved. This high conversion efficiency is gained through the combined effects of plasmonic photocatalysis accompanied by the acoustic-activated CO_2_ conversion mechanisms. The photonic mechanism of CO_2_ conversion can be explained based on the generation of hot electrons at plasmonic heterointerfaces (Figure 1e). In these 2D heterointerfaces, the plasmonic-generated hot electrons are transferred to the adjacent 2D Ga_2_O_3_ semiconductor to enable the conversion of adsorbed CO_2_ into O_2_ and carbon. The triboelectric forces accompanied by plasmonic nanostructures can activate the mechanism of C=O debonding and then enable the conversion of CO_2_ atoms into value-added byproducts [31,75]. Therefore, the photogenerated hot electrons at plasmonic 2D heterointerfaces between Ag and Se nanodomains and 2D Ga_2_O_3_ nanosheets have provided the excess electrons for the CO_2_ conversion through these reactions:

(7) $$\mathrm{Electron}\;(e^{-1})+{\mathrm{CO}}_{2}\to \;\mathrm{hole}\;(h^{+})+CO_{2}^{{}^{\circ}-}$$

(8) $$CO_{2}^{{}^{\circ}-}+e\;\to \;\mathrm{CO}\;+\;h^{+}\;+\;O^{2-}$$

(9) $$\mathrm{CO}+2\;e\;\to \;C\;+\;2h^{+}\;+\;O^{2-}$$

(10) $$2O^{2-}\;\to \;O_{2}\;+\;4e^{-}$$

In these reactions, the plasmonic–triboelectric generated electron transfer to CO_2_ (Equation (7)). The following electron transfer to C$O_{2}^{{}^{\circ}-}$ radicals was accompanied by the generation of CO and O^2−^ radicals (Equations (8) and (9)). The CO later turns into carbon-based materials, while the O^2−^ turns into O_2_ after reduction (Equation (10)) [75]. We further investigated the CO_2_ conversion efficiency of 2D Ga_2_O_3_-Se nanostructures. Interestingly, in the similar conditions, the CO_2_ conversion efficiency of 2D Ga_2_O_3_-Se nanostructures was less than that of 2D Ga_2_O_3_-Ag nanostructures after 30 min of cyclic reactions in reactor. Despite the lower efficiency, the 2D Ga_2_O_3_-Se nanostructures still present a high CO_2_ conversion efficiency (higher than 92%) (Figure 5e). In the case of 2D Ga_2_O_3_-Se nanostructures, the effect of piezoelectric Se nanodomains should also be considered. The piezoelectric CO_2_ conversion was recently investigated in several studies. In this mechanism, it is expected that the 2D Ga_2_O_3_-Se heterointerfaces provide extra electrons to break the strong *sp* hybridization of CO_2_ atoms and activate the piezocatalytic conversion of CO_2_ into value-added by products [76]. The high-frequency ultrasound waves continuously polarize the 2D Ga_2_O_3_-Se heterointerfaces and establish the built-in electric field at the 2D piezoelectric structure. Consequently, electron–hole pairs are separated continuously from each other and move to opposite surface of piezoelectric material [76]. We further collected the carbon-rich byproducts of reactions to calculate their production rate. After stabilization of CO_2_ conversion in the chamber containing 2D Ga_2_O_3_-Ag nanosheets, the amount of ~180 μmol/g^−1^ of solid carbon was extracted after 30 min of catalysis reaction, which is equal to the carbon production rate of ~360 μmol g^−1^h^−1^ (Figure 5f). This number is higher than the conversion rate of most of the previously reported conversion performance of 2D-based photocatalysts for CO_2_ reduction (Table 1).

**Table 1:** A list of 2D materials employed for CO_2_ conversion process.

Materials	Source of Energy	Conversion Product & Rate	Ref.
Ga_2_O_3_-Ag	250 W Xenon-lamp + ultrasonic (20 Hz, 380 W)	Solid carbon; ~360 μmol g^−1^h^−1^	Present
TiO_2_ nanosheets	300 W Hg lamp	HCOOH; 1.9 μmol g^−1^h^−1^	[77]
TiO_2_ nanosheets/graphene	300 W Xe lamp	CO; 52.3 μmol g^−1^h^−1^	[78]
SnS_2_/TiO_2_ nanosheets	300 W Xe lamp	CH_4_; 23.0 μmol g^−1^h^−1^	[79]
Cu_2_O octahedrons/WO_3_ nanoflakes composite	300 W Xe lamp	CO; 3.45 μmol g^−1^h^−1^	[80]
ZnV_2_O_6_ nanosheet/RGO nanosheet	35 W HID Xe lamp	CH_3_OH; 515.4 μmol g^−1^h^−1^	[81]
Graphene bridged ZnV_2_O_6_/pCN nanosheets	35 W Xe lamp	CH_3_OH; 542.92 μmol g^−1^h^−1^	[82]
BiOBr nanosheets	300 W Xe lamp	CO; 4.45 μmol g^−1^h^−1^	[83]
Bi_4_O_5_Br_2_ nanosheet	300 W Xe lamp	CO; 31.57 μmol g^−1^h^−1^	[84]
BiOBr nanosheets with surface Bi vacancies	300 W Xe lamp	CO; 20.1 μmol g^−1^h^−1^	[85]
MoS_2_-nanosheets/TiO_2_-nanosheets	300 W Xe lamp	CH_3_OH 10.6 μmol g^−1^h^−1^	[86]
Cs_2_SnI_6_/SnS_2_ nanosheet	100 W Xe lamp	CH_4_; 6.09 μmol g^−1^h^−1^	[87]
Ni metaleorganic framework monolayers	5 W white LED light	CO; 12,500 μmol g^−1^h^−1^	[88]
CeO_2_/Ti_3_C_2_	350 W Xe lamp	CO; 40.2 μmol g^−1^h^−1^	[89]
CsPbBr_3_/Ti_3_C_2_Tx	300 W Xe lamp	CO; 26.32 μmol g^−1^h^−1^ CH_4_ 7.25 μmol g^−1^h^−1^	[90]

## 4. Conclusions

In summary, Ag and Se plasmonic polycrystalline nanodomains were grown on the surface of 2D Ga_2_O_3_ catalyst via sonochemical assisted synthesis. These 2D heterointerfaces were found highly efficient platforms for plasmonic CO_2_ photocatalysis in the presence of mechanical energies of acoustic waves. It was observed that the triboelectric energy accompanied by the plasmonic photocatalysis co-contributed to enhance the CO_2_ conversion efficiency to values higher than 94%. The challenging process of growth of plasmonic Ag and Se nanodomains was crucially dependent on the precursor selection as well as synthesis. The material characterization studies showed the polycrystalline nature of Ag and Se nanodomains grown on the surface of 2D Ga_2_O_3_ nanosheets. The AFM studies further confirmed the uniform distribution of plasmonic nanodomains on the surface of 2D nanostructures. PL spectroscopy further confirmed the local field enhancement and surface plasmon resonance (SPR) interactions of Ag and Se nanodomains on 2D Ga_2_O_3_ nanosheets. The enhanced CO_2_ conversion capability of these nanostructures originated from the following factors: the plasmonic photocatalysis at Ga_2_O_3_-Ag and Ga_2_O_3_-Se heterointerfaces, the plasmonic hot-electron transfer at catalyst interfaces, and finally the acoustic-activated CO_2_ debonding and conversion. Consequently, this principally developed novel technique for solar-activated acoustic photocatalysis of CO_2_ into value-added byproducts provides excellent opportunities for establishment of technological platforms for generation of clean fuels similar to O_2_ through solar-assisted conversion of greenhouse gases.

## Data Availability

Available upon request from authors.
